# Hyperglycemia Associated Metabolic and Molecular Alterations in Cancer Risk, Progression, Treatment, and Mortality

**DOI:** 10.3390/cancers11091402

**Published:** 2019-09-19

**Authors:** Pranay Ramteke, Ankita Deb, Varsha Shepal, Manoj Kumar Bhat

**Affiliations:** National Centre for Cell Science, Savitribai Phule Pune University, Ganeshkhind, Pune-411 007, India; pranay1130@gmail.com (P.R.); Ankita.deb12@gmail.com (A.D.); varsha@nccs.res.in (V.S.)

**Keywords:** Hyperglycemia, diabetes, glucose, cancer, metabolism, risk, mortality, chemotherapy

## Abstract

Cancer and diabetes are amongst the leading causes of deaths worldwide. There is an alarming rise in cancer incidences and mortality, with approximately 18.1 million new cases and 9.6 million deaths in 2018. A major contributory but neglected factor for risk of neoplastic transformation is hyperglycemia. Epidemiologically too, lifestyle patterns resulting in high blood glucose level, with or without the role of insulin, are more often correlated with cancer risk, progression, and mortality. The two conditions recurrently exist in comorbidity, and their interplay has rendered treatment regimens more challenging by restricting the choice of drugs, affecting surgical consequences, and having associated fatal complications. Limited comprehensive literature is available on their correlation, and a lack of clarity in understanding in such comorbid conditions contributes to higher mortality rates. Hence, a critical analysis of the elements responsible for enhanced mortality due to hyperglycemia-cancer concomitance is warranted. Given the lifestyle changes in the human population, increasing metabolic disorders, and glucose addiction of cancer cells, hyperglycemia related complications in cancer underline the necessity for further in-depth investigations. This review, therefore, attempts to shed light upon hyperglycemia associated factors in the risk, progression, mortality, and treatment of cancer to highlight important mechanisms and potential therapeutic targets.

## 1. Introduction

Cancer and diabetes together account for high mortality globally. Existing evidences suggest a significant role of hyperglycemia in all facets of cancer which has been repeatedly overlooked, from oncogenesis to mortality [1,2,3,4,5,6,7,8]. Hyperglycemia is defined by an elevated glucose level in blood plasma (>125 mg/dL while fasting and >180 mg/dL 2 h postprandial) [9]. Hyperglycemia occurs due to various reasons such as diabetes mellitus type I and type II, obesity, stress, pancreatic failure, drugs such as glucocorticoids, and estrogen. Numerous studies have correlated hyperglycemia with an increased risk of cancer [10,11]. Patients consuming foods with a high glycemic index have an enhanced cancer risk [12]. Women in the highest quartile for blood glucose levels have an increased risk of breast cancer compared to women in the lowest quartile (RR 1.63; 95% CI) [13]. While many cohort studies have highlighted diabetes-associated cancer risks, only a few reports have assessed the underpinning effect of hyperglycemia on the risk of cancer. The Vasterbotten intervention project, comprising 33,293 women and 31,304 men, calculated the relative risk of cancer due to hyperglycemia after adjusting for age, recruitment year, time of fasting, and smoking status for 10 years of repeated measurements. The study clearly demonstrated that total cancer risk in women increased with higher plasma level of post-load and fasting glucose. Also, elevated fasting glucose levels in men and women were associated with statistically significant risks of pancreatic cancer, malignant melanoma, endometrial, and urinary tract cancers. Body mass index (BMI) adjustments showed no significant difference in risk estimates, suggesting that a high blood glucose level—irrespective of obesity or diabetes—is likely to be the key player in enhancing cancer risk [14]. HbA1c, a long-term marker of blood glucose level, was correlated with an increased all site cancer risk in a study comprising 29,629 patients. Higher levels of HbA1c, even within the non-diabetic range, were associated with 28% higher risks of almost all cancers. However, in the case of liver cancer, low HbA1c levels were correlated with higher cancer risk [15]. Also, glucose tolerance was associated with an increased all site cancer incidence in a 20 year cohort study of men and women [16]. The risk of premalignant lesions, which act as precursors for cancer, is higher in hyperglycemic patients [17].

Hyperglycemia increases the risk of cancer and contributes towards its progression and mortality. Various cancers advance more aggressively under hyperglycemic conditions—especially cancers of the liver, pancreas, mammary gland, and endometrium [18,19]. In a meta-analysis of eight studies comprised of 4342 patients, hyperglycemia was associated with adverse disease-free survival (DFS) and overall survival (OS) of cancer patients [20]. Despite several reports stating an enhanced risk, progression, and mortality of cancer due to hyperglycemia, literature about the possible metabolic and molecular events responsible for creating a window of neoplastic transformation is scarce.

## 2. Hyperglycemia and Risk Factors for Cancer

**Figure 1:**
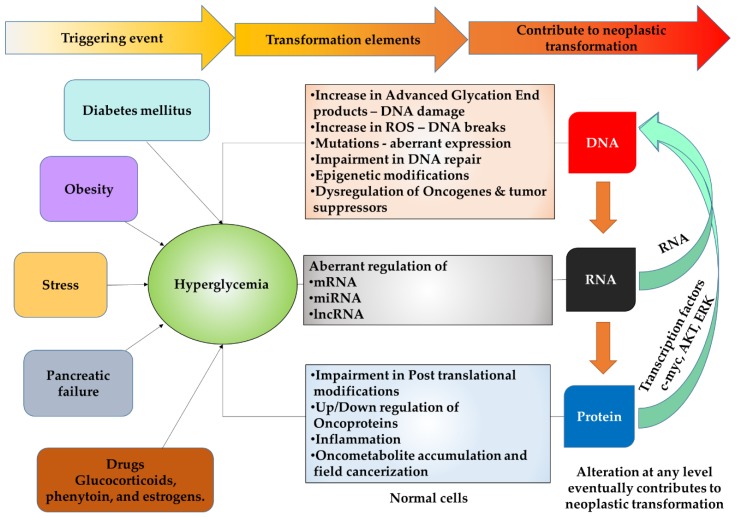
Hyperglycemia associated risk factors for cancer.

Increased blood glucose levels affect the normal cellular system majorly at three steps 1. DNA (Genetic) 2. RNA (Transcription), and 3. Protein (Translation), which may contribute to dysregulated growth (Figure 1).

### 2.1. Hyperglycemia and DNA Damage

DNA damage plays a pivotal role in carcinogenesis [21]. Hyperglycemia directly or indirectly causes DNA damage, ROS formation, DNA breaks, mutation accumulation, impairment in DNA repair, and dysregulation of oncogenes and tumor suppressors via various mechanisms.

It promotes the formation of glycated moieties, such as advanced glycation end (AGE) products in various tissues [22]. AGE are products of a non-enzymatic reaction between reducing sugars and the amino groups of nucleic acids, lipids, or proteins. Accumulation of AGE and its precursors can lead to DNA damage by reacting with DNA bases and inducing ROS, NFkB, the receptor for AGE (RAGE), or inflammation, thereby contributing to carcinogenesis, e.g., pancreatic cancer and hepatocellular carcinoma (HCC) [23,24,25]. Excess ROS generated by AGE accumulation promotes oxidation of DNA bases, especially of guanine, resulting in G to T transversions. Oxidized bases, if not repaired, lead to mutations which can trigger oncogenes or deactivate tumor suppressor genes, causing initiation and/or progression of different cancers [26,27]. Methylglyoxal induced AGE accumulation in tissues may occur even in a relatively mild diabetic condition and cause DNA damage [28]. Avoiding sweetened foods and drinks is often recommended for inhibiting glycation product formation and accumulation, thereby highlighting the role of hyperglycemia in DNA damage [24].

Hyperglycemia also causes DNA damage by altering oncogene/tumor suppressor expression. Proximal tubular epithelial cells under high glucose conditions and type 1 diabetes-induced hyperglycemia undergo excess *AKT* oncogene activation. This further leads to 8-oxodG accumulation, a marker of oxidative DNA damage in vitro and in vivo models [29]. High glucose also induced phosphorylation of p53 at ser 18 in ventricular myocytes, which is indicative of DNA damage [30].

In addition, hyperglycemia also increases the accumulation of mutations in DNA. If the mutations induced are in oncogenes or tumor suppressors, it can contribute to elevated cancer risk. Diabetic mice exhibit increases in a number of mtDNA mutations and mutation sites in oocytes [31]. Moreover, diabetic patients have a higher incidence of somatic transversion mutations in mtDNA [32]. Hyperglycemia-induced mutations increased the mortality of subjects with DNA damage, which predisposed to cancer. In a meta-analysis of 2,645,249 subjects, patients with preexisting Diabetes mellitus (DM) had increased all-cause mortality risk in women with BrCa alteration by 37% (HR = 1.37; 95%CI: 1.34–1.41; *p* = 0.02) [33]. In oral oncogenesis, increased accumulation of mutations in the p53 gene occurs under diabetic conditions, leading to enhanced proliferation of tumor cells [34]. Moreover, in endothelial cells, high glucose levels induce DNA breaks, thereby contributing to neoplastic transformation [35]. Excess glucose metabolism in β cells cause double-strand breaks in DNA and activate p53 and apoptosis, possibly via oxidative stress and ROS generation [36]. High glucose enhances the number of micronuclei, nucleoplasmic bridges, and nuclear buds in normal colon cells in folate-deficient conditions, hence contributing to genomic instability [37].

Hyperglycemia causes DNA alterations, and the genes responsible for diabetes risk are also associated with an increased risk of cancer. The long island breast cancer study revealed that the genetic polymorphisms which account for an increased diabetes risk are involved in enhanced mortality and risk of developing breast cancer; for example, *SLC30A8* (a zinc transporter insulin-related secretion gene), *CDKN2A-CDKN2B* (cell cycle related genes), *IGFBP2* and *IRS2* (Insulin pathway related genes). The single nucleotide polymorphisms (SNPs) listed indirectly suggest an association between genes involved in metabolic and molecular glucose signaling, the cell cycle, and risk/progression of cancer [38]. Type 2 diabetes (T2D) associated SNPs are also present in *JAZF1*, which plays an important role in stromal cancer oncogenesis. *JAZF1* downregulation impairs *AMPK* oncogene phosphorylation, thus demonstrating that aberrant *JAZF1* expression and SNPs links to oncogenesis and T2D pathogenesis. Moreover, *JAZF1* overexpression in C2C12 normal myoblast cells exhibited enhanced proliferation by altering *AMPK* expression. Collectively, these studies highlight the important role of hyperglycemia in DNA damage and neoplastic transformation [39].

Hyperglycemia also interferes with DNA repair mechanisms [40,41,42], which has been reported widely as the origin of carcinogenesis [43,44,45,46,47,48]. Hyperglycemic conditions significantly reduce the functionality of DNA repair mechanisms by downregulating DNA damage repair genes. If normal cells are not able to maintain genomic stability, neoplastic transformation is favoured. In a rat prostate model and normal human prostatic RWPE-1 cell line, a number of DNA damage repair genes such as *Rad51L3*, *Mre11*, *Xrcc3*, *dpoLL*, *Nudt5*, *Ube2c*, *Msh5*, *Msh3*, and *SEMA6c* are downregulated under diabetic conditions [42]. Nucleotide excision repair is regulated by xeroderma pigmentosum complementation group D protein (XPD), which was downregulated in high glucose conditions in Chinese hamster ovary (CHO) cells [49]. Moreover, DNA damage repair genes were downregulated in peripheral blood mononuclear cells (PBMC) isolated from diabetic patients (n = 20) as compared to their normal counterparts (n = 8) [50]. These reports state the crucial role of hyperglycemia in interfering with DNA damage repair.

Besides directly affecting genetic stability, hyperglycemia also causes epigenetic dysregulation, leading to a series of downstream signaling cascades, which, in turn, increases the risk of neoplastic transformation [51].

### 2.2. Hyperglycemia and RNA

Hyperglycemia causes transcriptional modifications in cells by affecting mRNA, transcription factors, miRNA, and lncRNA. Transcription factors are regulators of mRNA expression in tissues. Carbohydrate responsive element binding protein (ChREBP) is a promoter of glycolysis in normal and cancer cells. Excess glucose causes a hepatocyte nuclear factor 4 (HNF4) mediated increase of ChREBP transcription factor [52]. High glucose treated human umbilical vein endothelial cells (HUVEC) elicited upregulation and accumulation of Alu-sc dsRNA leading to increased oxidative stress by promoting ROS generation and suppressing eNOS and SOD2 at both transcriptional and translational levels [53]. In another study, hyperglycemia altered the expression of hypermethylated in cancer 1 (HIC1) and increased ROS accumulation in renal tubular epithelial cells (HK-2) by epigenetically repressing *SIRT1* [54].

Recent reports suggest the emerging roles of miRNA and lncRNA in several functions at a sub transcriptional level. miRNA and lncRNA provide an additional layer of regulatory control in cellular functioning, and hence, their altered expression plays a pivotal role in transformation and dysregulated signaling [55]. *OncomiR-9* is overexpressed in prediabetic patients and progressively enriched in T2D patients. It induces immortalization/transformation of normal mouse bone marrow progenitor cells in vitro and promotes leukemogenesis in vivo [56,57]. Similarly, miR-199a-5p downregulates hypoxia-inducible factor-1 (*HIF-1α*) oncogene and oxidative stress-induced growth inhibitor 2 (*OSGIN2*) expression. *miR-199a-5p* is downregulated under diabetic conditions, and its expression is directly correlated to the prognosis of soft-tissue sarcoma patients [58,59].

MALAT1 lncRNA expression is associated with carcinogenesis of different cancers [60,61]. Interestingly, it was overexpressed in streptozotocin (STZ) induced diabetic mice, upregulated in the diabetic mice retinas, in the RF/6A hyperglycemia model, in aqueous humor samples, and the epiretinal fibrovascular membranes (FVM) of diabetic patients [62,63,64]. Another lncRNA, ANRIL, is upregulated under high glucose conditions in normal cells and is correlated with the carcinogenesis of gastric, oral, breast, and cervical cancers [65,66,67,68,69]. Altogether, these evidences demonstrate that hyperglycemia affects cells at transcriptional as well as sub-transcriptional levels, predisposing them to neoplastic transformation.

### 2.3. Hyperglycemia and Proteins

Hyperglycemia contributes to carcinogenesis by triggering various oncogenic pathways via inflammation, oncometabolite accumulation, post-translational modifications, proto-oncogene dysregulation, and field cancerization. Oncometabolites such as fumarate accumulated more under hyperglycemic conditions. Fumarate accumulation can trigger oncogenesis and drive transformation, even without genetic alteration [70]. Diabetic patients had altered YAP/TAZ-TEAD signaling in normal mucosa, which play key roles in the initiation and field cancerization of the colon compared to non-diabetic patients [71]. Hyperglycemia also causes inflammation [72] via increasing obesity [73], gut permeability [74], and LDL levels in humans [75]. Inflammatory markers like NFKB, TNF-α, IL-6, and IL-18 levels were elevated in hyperglycemic patients [76,77,78,79]. Inflammation is strongly correlated with various cancers [80,81]. Besides these, almost all the major proto-oncogenes such as *c-myc* [82], *HIF-1α* [83], *AKT*, *mTOR* [84], *c-FOS*, and *c-JUN* [82] are activated/overexpressed under hyperglycemic conditions in normal cells. Although overexpression of a single molecule may not lead to carcinogenesis, it contributes towards the larger process which involves a constellation of steps.

Post-translational modifications (PTM’s) are specific changes in proteins at pre-coded sites that dictate their activity for different functions like protein synthesis, cell proliferation, apoptosis, etc. Several PTM’s such as phosphorylation, ubiquitination, SUMOylation, and acetylation play a decisive role in cancer risk and incidence. The important PTM’s in cancer are those involved in chromatin histone modifications and proliferation/cell cycle. Tumor cells have a euchromatin structure, and much of their DNA is unpacked and active, owing to the acetylation of histone proteins which exposes DNA to transcription factors (TF). Hyperglycemia inhibited phosphorylation of *TET2* (a tumor suppressor), resulting in its destabilization, and dysregulation of its tumor suppressive activity and its substrate, 5-hydroxymethylcytosine (5hmC), in vitro as well as in vivo. 5hmC is often decreased in many cancers and is linked with DNA demethylation and gene activation/deactivation. The anti-diabetic drug metformin shields phosphorylation of *TET2*, thereby increasing its stability and 5hmC levels. This study clearly demonstrates how high extracellular glucose levels lead to a series of global epigenetic, genetic, and molecular alterations, resulting in an oncogenic state [85]. Pyruvate kinase M2 (PKM2), a glycolysis enzyme, phosphorylates histone H3 causing dissociation of HDAC3 from CCND1 and the *MYC* promoter region, thereby increasing their expression, tumor cell proliferation, cell cycle progression, and brain tumor formation. Also, histone H3 phosphorylation levels correlate with nuclear expression levels of *PKM2*, malignancy grades of glioma, and nasopharyngeal carcinoma and its prognosis [86,87]. It is important to note here that glucose regulates the expression of *PKM2* transcriptionally as well as translationally via Sp1 [88].

## 3. Hyperglycemia and Cancer Progression

Hyperglycemia affects the following characteristics in cancer progression: (a) Metabolic reprogramming and molecular alterations; (b) Avoiding immune destruction, increasing tumor, and promoting inflammation; (c) Proliferation and apoptosis inhibition; (d) Metastasis.

### 3.1. Metabolic Reprogramming and Molecular Alterations

**Figure 2:**
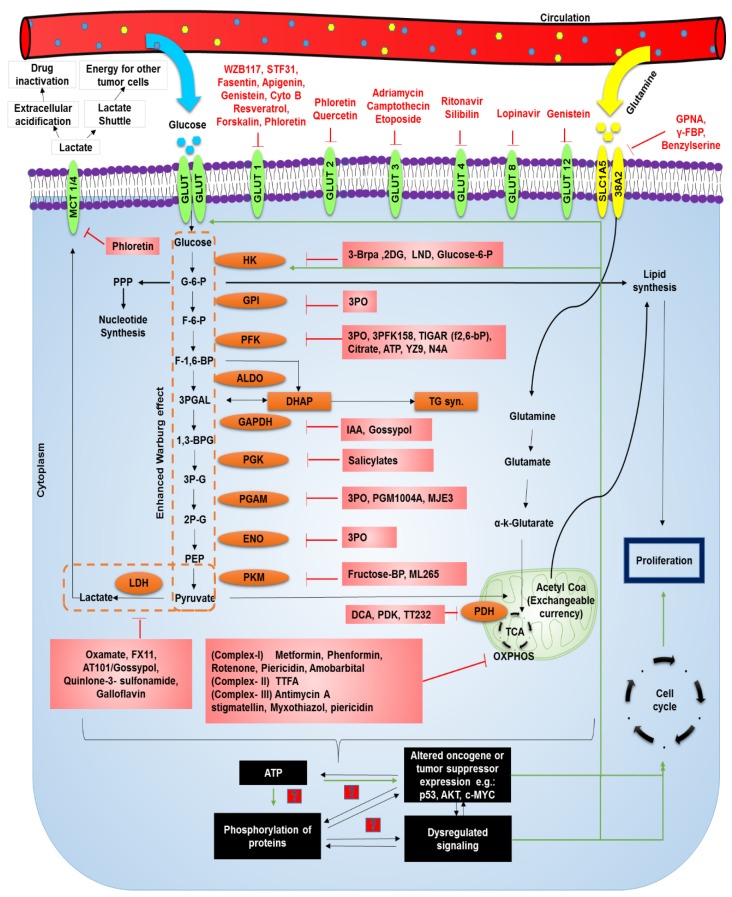
Hyperglycemia associated metabolic reprogramming in cancer cells and potential targets. Pathways altered due to hyperglycemia leading to proliferation are indicated by green arrows. Inhibitors of various molecules are indicated in red. The inhibitors presented here and the corresponding clinical or research studies are mentioned in Table 1 with their respective targets. STF31: 4-[[[[4-(1,1-Dimethylethyl)phenyl]sulfonyl]amino]methyl]-N-3-pyridinyl-benzamide; CYTO B: Cytochlasin B; GPNA: L-γ-Glutamyl-p-nitroanilide; FBP: Fructose-1,6-bisphosphate; GLUT: Glucose transporter; MCT: Monocarboxylate transporter; PPP: Pentose Phosphate Pathway; G6P: Glucose-6-phosphate; F6P: Fructose-6-phosphate; F1,6BP: Fructose-1,6-bisphosphate; 3PGAL: Glyceraldehyde-3 phosphate; 13BPG: 1,3 Bisphosphoglyceric acid; 3PG: 3-phosphoglycerate; 2PG:2-phosphoglycerate; PEP: Phosphoenol pyruvate; HK: Hexokinase; GPI: Glucose-6 phosphate isomerase; PFK: Phosphofruktokinase-1; ALDO: Aldohexose; DHAP: Dihydroxyacetone phosphate; GAPDH: Glyceraldehyde 3-phosphate dehydrogenase; PGK: Phosphoglycerate kinase; PGAM: Phosphoglycerate mutase-1; ENO: Enolase; PKM: Pyruvate kinase M1/M2; TG: Triglyceride; 3BRPA: 3 Bromopyruvic Acid; 2DG: 2-deoxyglucose; LND: Lonidamine; 3PO: (2E)-3-(3-Pyridinyl)-1-(4-pyridinyl)-2-propen-1-one; TIGAR: TP53-inducible glycolysis and apoptosis regulator; ATP: Adenosine triphosphate; IAA: 1-O-Indol-3-ylacetyl-beta-D-glucose; DCA: Dichloroacetic acid; PDK: Pyruvate Dehydrogenase kinase; LDH: Lactate Dehydrogenase; OXPHOS: Oxidative phosphorylation.

One of the most catastrophic hallmarks of cancer cells is its ability to reprogram metabolism—including that of glucose. Normal metabolism generates energy that meets regular functioning. However, owing to excess energy demands, cancer cells shift to an inefficient glycolytic mode by directing a major flux of nutrients into glycolysis instead of oxidative phosphorylation (OXPHOS); this is known as the Warburg effect. It was previously hypothesized that cancer cells reprogram their metabolism due to defects in mitochondria. However, it was later proven that not only are mitochondria functional in many cancers, but they also contribute as a major source of energy. By upregulating glycolysis, cancer cells increase the production of glycolytic intermediates, which function as important precursors required for the synthesis of carbohydrates, fats, and proteins [89]. They thereby prevent the accumulation of NADH and inhibit the feedback loop of ATP [90]. This facilitates the production of ATP at a much faster rate, even in the presence of oxygen [91]. For this purpose, cancer cells uptake more glucose as can be detected by positron emission tomography (PET) than normal cells, which gives a selective advantage in a nutrient limiting environment [92]. However, in hyperglycemia, these restrictions are warded off as glucose is abundantly available. Hyperglycemia, therefore, promotes glycolysis in various cancer cells [93,94]. It increases the expression of glycolytic enzymes such as hexokinase-II (HK-2) and pyruvate kinase M and contributes to enhanced metabolic reprogramming [95]. Also, lactate generated as a byproduct of this rewiring is utilized as a shuttle and energy source for tumor cells in regions where blood and oxygen cannot reach due to inefficient angiogenesis via transporters such as MCT1 which can be targeted by AZD3965 [96,97] (Figure 2).

Excess ATP generated via rewiring is utilized for a series of dysregulated signaling events contributing to the loss of cell cycle regulation and uncontrolled proliferation [98]. How cancer cells activate or regulate favorable pathways and control cell cycle—despite the presence of gatekeepers and strict regulatory checkpoints—by using excess ATP derived from metabolic reprogramming is an intriguing topic for further in-depth investigation.

For upregulation of metabolism, molecules such as GLUTs, and their translocation to the membrane—together with upregulation of glycolytic enzymes—are essential. These events take place under the aberrant expression of oncogenes/tumor suppressors such as *PI3K*, *AKT*, *p53*, and *RAS* [90]. However, the activation of potential oncogenes and tumor suppressors can occur only subsequent to an increase in ATP production. The GLUT family of receptors are often upregulated in different cancers; GLUT-1 is upregulated in a number of cancers [99], GLUT-2 in hepatocellular carcinoma (HCC) [100], GLUT-3 in endometrial cancer [101], GLUT-4 in prostate cancer [102], GLUT 8 in breast cancer [103], and GLUT 12 in breast and prostate cancer [104]. Numerous small molecule inhibitors and chemical approaches have been devised to block these receptors as strategies to treat cancers (Table 1). 

Following the entry into the cells, glucose is metabolized via hexokinase into glucose-6-phosphate (G-6-P) which is utilized for either glycolysis, nucleotide synthesis, or lipid synthesis, all of which are upregulated in cancer. Strategies for targeting enzymes which regulate key metabolic steps have been devised (Table 1). 

Excess lactate produced due to metabolic shift is effluxed out of cells via the monocarboxylate 4 (MCT-4) class of transporters. This creates an acidic tumor microenvironment causing extracellular drug deactivation. Since the accumulation of lactate induces a metabolic catastrophe, inhibition of extracellular efflux of lactate via MCT-4 inhibitors such as phloretin have been conceived to achieve intracellular acidic cytotoxicity. In addition to glucose, cancer cells rely on glutamine as an alternate energy source, which enters into Krebs cycle and is used for the production of scavenging molecules to keep excess ROS in check. In mouse model studies, glutamine uptake has also been reported to increase in diabetic conditions. Therefore, glutamine metabolism has been targeted in cancer treatment by the inhibition of glutamine transporter, SLCA15, by L-γ-glutamyl-p-nitroanilide (GPNA), γ-2-fluorobenzyl proline (γ-FBP), benzyl serine, aminooxetanecarboxylate (AOC), and chloroalanine [105,106] (Table 1). 

Also, cancer cells exhibit enhanced lipogenesis, which is tightly coupled with glucose metabolism. Hyperglycemia enhances the expression of ChREBP in cancer cells, which is a known promoter of lipogenesis [107]. Many tumor types produce 95% of mono-unsaturated and saturated fatty acids (FA) de novo, even in the presence of an excess dietary lipid supply. It is utilized for survival during oxidative stress, resistance towards drugs, signal transduction, gene expression, and rapid proliferation, in addition to forming building blocks for the synthesis of membrane phospholipids. This is achieved by utilizing energy derived from metabolic reprogramming in overexpression of key molecules involved in lipid metabolism such as FASN, acetyl-CoA-carboxylase (ACACA), and ATP-citrate lyase (ACLY). In leukemia and prostate tumors, cancer cells exhibit a major dependence on lipids as the primary source of energy. As these molecules play a major role in lipid metabolism, which is correlated with a poor prognosis in various cancers, they have been proposed as important anticancer targets via drugs such as TVB-2640, orlistat, soraphen A, cerulenin, etc. However, upon inhibition of lipogenesis or FA synthesis, cancer cells also possess the ability to utilize extracellular lipids via lipolysis [108,109].

Along with lipogenesis, cancer cells also upregulate nucleotide synthesis via metabolic rewiring. Hyperglycemia can drive metabolism towards the pentose phosphate pathway (PPP) because of activation of oncogenes and tumor suppressors such as *c-MYC* and *mTOR*. It enhances the production of purine nucleotides in cancer cells [110]. As nucleotide synthesis is critical for rapid proliferation, this pathway has been targeted widely via inhibition of important intermediates or enzymes such as DHFR (methotrexate, pemetrexed), glucose-6-phosphate dehydrogenase (polydatin), thymidylate synthase (Pemetrexed, capeciatbine, 5-fluorouracil), PRPP amidotransferase (6-mercaptopurine, 6-thioguanine), DNA polymerase/ribonucleotide reductase (gemcitabine, cytarabine), dihydroorotate dehydrogenase (leflunomide), etc. [111,112,113,114,115,116,117].

### 3.2. Avoiding Immune Destruction and Increasing Tumor Promoting Inflammation

Evidence suggests that hyperglycemic conditions increase cancer-associated inflammation by the secretion of cytokines. High glucose stimulates the upregulation of TNF-α, IFN-γ, resistin, and IL-6 [79]. Hyperglycemia, in obese patients, elevates pro-inflammatory cytokines, and tumor promoted inflammation. These cytokines are also responsible for insulin resistance [118,119,120] and activation of oncogenic downstream signaling pathways such as NFKB, c-Jun, and JNK/MAPK [120,121,122]. Pathologies associated with these cytokines are mitochondrial dysfunction, oxidative stress, intracellular lipid accumulation in the liver or skeletal muscle, and decreased β-oxidation, which are all linked to cancer progression, as shown in Supplementary Figure S1 [123].

Several immune cells exert an impact on tumor progression and prognosis. Hyperglycemia has a profound effect on these cells. Tumor-infiltrating leukocytes—such as CD8+ T cells, neutrophils, MDSCs, macrophages, etc.—are dysregulated under the influence of high blood glucose levels. Hyperglycemia induces the Hexosamine biosynthetic pathway (HBP) by enhancing M2 polarization, resulting in an upregulation of O-GlcNacylation [124]. Myeloid derived suppressor cells (MDSCs) are reported to be present at tumor sites, suppressing anti-tumor immunity—particularly T cells through multiple mechanisms. SIRT1 plays a key role in regulating MDSC differentiation to M1 and M2 phenotypes through HIF-1α induced metabolic reprogramming. This impacts MDSC induced functions in immune suppression and tumor growth [125,126]. Moreover, high glucose stimulates monocytes and macrophages to enhance the secretion of IL-6 by inducing PKC and TNF-α, which promote tumor progression and invasiveness [127,128,129,130]. An increase in glucose metabolism leads to low CD8+ T cell infiltration in renal cell carcinoma, which attenuates the mTOR pathway and IFNγ production [131,132]. In melanoma, the impaired IFNγ expression in tumor-infiltrating T cells and NK cells, along with LDHA activity associated lactate production, promotes tumor growth by exerting an immunosuppressive phenomena [133]. Altogether, hyperglycemia and its effect on these altered phenomena significantly affect tumor progression and presents a potential avenue for therapeutic intervention.

### 3.3. Proliferation and Apoptosis Inhibition

Normal cells grow slowly and exhibit apoptosis under hyperglycemic conditions [134,135,136]. However, this phenomenon is reversed in cancer. Hyperglycemia fuels the excess energy required for rapid cell proliferation. Cancer cells proliferate faster with negligible apoptosis in high glucose conditions in vitro as well as in vivo [137]. Studies performed in several cancer cells demonstrate that hyperglycemia and obesity favor cancer cell proliferation by oncogene or metabolic and molecular alterations [118,138]. Hyperglycemia promotes cancer progression by independent and synergistic mechanisms. Epidermal Growth Factor and its receptor, EGFR, were upregulated under hyperglycemic conditions in pancreatic cancer [139]. High glucose stimulates Protein kinase C (PKC) and Peroxisome proliferator-activated receptor gamma (PPARγ) levels, which induced an aggressive phenotype [140]. In diabetes-associated inflammation, several factors which enhance cancer progression—like peripheral estrogen, pro-mitogen cytokines, and growth factors—were increased [84]. Hyperglycemia promotes breast cancer progression by altering leptin/IGFR1 and Akt/mTOR signaling, whereas, in pancreatic cancer, it contributes to ROS stimulated cancer progression via suppression of the JNK and c-Jun pathways [141].

Apoptosis inhibition, an important characteristic in transformation, is improperly regulated in cancer cells. High glucose conditions triggered apoptosis in normal cells. However, it protects cancer cells from cytochrome-c mediated apoptosis [142]. Protein Kinase C (PKC) dependent ubiquitin-proteasome activation in high glucose increased proliferation and prevented apoptosis in breast cancer cells [143]. Metformin rescues breast cancer cells from apoptosis by suppressing STAT3 and Bcl-2, and elevating Bax levels [144]. Hyperglycemia inhibits the expression of growth arrest-specific 5 (*GAS5*) and subsequently elevates tribbles homolog 3 (*TRIBS3*), which inhibits apoptosis and induces proliferation of non-small cell lung cancer [145].

### 3.4. Metastasis

Metastasis causes an aggressive spread of cancer to other body parts. Studies over a period have shown hyperglycemic conditions enhance the migration of cells and reengineers them into primary lesions. Hyperglycemic cancer patients have a higher proportion of metastasis and worse outcomes compared to patients without hyperglycemia [18]. In pancreatic cancer, hyperglycemia increased lymph node metastasis by 27.8% and liver metastasis when compared with euglycemic conditions (14.3%) [146]. 

The mechanisms by which high glucose aggravates migration of cancer cells are unclear. Hyperglycemia-induced ROS production promotes the motility and invasiveness of cancer cells. Oxidative stress also induces epithelial to mesenchymal transition (EMT) and vascular destruction [18]. TGF-β/PI3/AKT pathways and upregulated *HO-1* expression, which enhances tumor invasion, were induced under hyperglycemic conditions [147]. Moreover, hyperglycemia increases the migratory spread of cancer cells by impairing G-CSF secretion and hinders the mobilization of antitumor neutrophils [148]. Variation in the glycemic index in pancreatic ductal carcinoma alters the pro-metastatic signal axis, Rarb/Runx3/Col6a1, and promotes local invasion. Improper management of glucose levels in cancer patients may lead to increased metastatic seeding [149]. High glucose also aggravates cell migration by increasing the expression of global O-GlyNacylated proteins, vimentin, hexokinase, and glucosamine-fructose-6-phosphate amidotransferase (GFAT) [150].

## 4. Hyperglycemia and Treatment of Cancer

Hyperglycemia influences the outcome of cancer therapy via various mechanisms such as chemoresistance, drug deactivation, affecting drug pharmacokinetics and dosages, and impairing immune responses.

Hyperglycemia can result in chemo-toxicity and, on the contrary, impart chemoresistance in cancer cells. High glucose downregulates multidrug resistance protein 1 (MDR-1), thus conferring a selective advantage to 5FU and causing more cell death in MCF-7 breast cancer cells [151]. Contrastingly, hyperglycemia attenuated the anti-proliferative effect of chemotherapy. Docetaxel induced apoptosis was reduced by 40% for DU145 cells and 88% for LNCaP prostate cancer cells [152]. Hyperglycemia also decreases the efficacy of cancer drugs. In a meta-analysis of preclinical studies comprising of 14 cell lines and two animal models, the chemotherapeutic response was less in hyperglycemic (>15 mmol/L) conditions as compared to that in normoglycemic conditions (5 mmol/L). However, in 5 other cell lines, it was the opposite [153]. In gastric cancer, hyperglycemic conditions enhance NAMPT and Sirtuin 1 levels and upregulate mutated p53 expression and multidrug resistance (MDR) via p-glycoprotein (P-gp) [154]. Hyperglycemia promoted tumors display several aspects such as modulation in tumorigenic ability, enhanced glucose utilization by tumor cells leading to altered acidosis and drug deactivation, organ dysfunction, and dysregulation of *MDR-1*, *p53*, *Bcl-2*, etc. [155]. Organ dysfunctions impact the dosages tolerated by patients, increasing the risk of toxicity. Additionally, hyperglycemia also affects the pharmacokinetics of chemotherapeutic drugs. It increases the renal secretion of cisplatin, thereby lowering its circulatory concentration compared to the non-hyperglycemic condition [156].

Besides directly affecting cancer cells, hyperglycemia also impairs immune responses and contributes to ineffective immuno-chemotherapeutic regimes [157]. Chemotherapeutic drugs ipilumumab and pembrolizumab, along with immune checkpoint inhibitors like nivolumab (anti-PD1 or anti-CTLA-4), exhibit better progression-free survival in metastatic malignant melanoma when combined with anti-hyperglycemic drug metformin [158]. Numerous pre-clinical studies suggest that metformin and phenformin, along with immune checkpoint blockade agents, inhibit tumor cell metabolism by increasing endogenous CD8+ T cell metabolism as well as cytokine production, which regresses tumor progression by improving T cell functioning [158,159,160]. Metformin is also effective on CD19-CART cells, which inhibit cell proliferation, cytotoxicity, and induce apoptosis [161]. Resveratrol, a mimic of calorie restriction agent, inhibits cell proliferation, and tumor angiogenesis by increasing immunosurveillance mechanisms [162,163]. It functions as an immunomodulator and chemosensitizing agent in melanoma and neuroblastoma by improving IL-2 based immunotherapy [164,165]. These phenomena take place due to an increased infiltration of immune cells in the tumor microenvironment and by promoting the susceptibility of tumor cells to the cytotoxicity of killer cells activated by IL-2 [166]. Understanding how glucose levels modulate the activity of immune cells could facilitate designing effective therapeutic approaches and improve outcomes.

The mechanisms by which hyperglycemia exerts its effect on immuno-chemotherapeutic combination are least explored. Excess glucose aids tumor cells in escaping NK-mediated killing via regulation of MHC class I chain-related protein A/B(MIC A/B) [167]. CD8+ T cells depend on glucose availability for their clonal expression and exhibiting anti-cancer properties, which include cytolytic activity and cytokine secretion. Tumor progression and T cell effector functions are impaired by dysregulation of CD8+ T cell metabolism within the tumor microenvironment due to hyperglycemia. Several reports suggest that an administration of immune checkpoint blockade, along with anti-CTLA-4 or anti-PD-1, improves the glycolytic capacity and cytokine secretion from CD8+ T cells [131,168,169]. 

Because of rising concern, advancements have been made in cancer chemotherapy to overcome hyperglycemia-induced chemoresistance. An improved glycemic control may positively influence the patient therapeutic index. In MCF-7 breast cancer cells, Selenadiazole was used to overcome hyperglycemia-induced doxorubicin resistance via activation of the AMPK pathway [170]. Also, in breast cancer studies, hyperglycemia-induced chemoresistance can be partially reversed by inhibiting fatty acid synthase (FASN) or ceramide production [171].

Due to a complex interplay between hyperglycemia and cancer, antihyperglycemic drugs such as Thiazolidinediones (TZD) and bile acid sequestrants have often been devised alone or in combination with anticancer drugs for cancer treatment (Table 2). Metformin, an oral antidiabetic drug, has been widely investigated for anticancer therapy. Earlier studies have suggested that metformin used for T2DM treatment reduces the overall progression and mortality rate of cancer. It has been shown to reduce tumor formation in rodent animal models [172]. In spite of promising advances in this field, further investigations are required for detailed insights into hyperglycemia and its correlation with chemoresistance to improve patient outcomes. 

Hyperglycemia-induced effects, such as enhanced metabolic reprogramming in combination with dysregulated molecular signaling, contribute to uncontrolled proliferation; the same, in combination with chemoresistance or immune evasion, contributes to apoptosis inhibition. Thus, hyperglycemia, via a net result of uncontrolled proliferation and apoptosis inhibition, enhances cancer progression and mortality, as shown in Supplementary Figure S2.

## 5. Hyperglycemia and Cancer Mortality 

Hyperglycemia associated risk factors severely impact the mortality rate in cancer subjects. The correlation between increased random blood glucose (RBG) in non-diabetic breast cancer subjects with their overall survival (OS) and time to tumor recurrence (TTR) was analyzed. Patients with elevated random blood glucose levels were reported to have shorter OS (HR 3.01; 95% CI (1.70–5.33); *p* < 0.001) and TTR tumor reoccurrence rate (HR, 2.08; CI (1.04–4.16); *p* = 0.04) when compared to patients with non-elevated RBG levels after controlling for tumor grade, tumor stage, race and BMI (HR, 3.50; CI (1.87–6.54); *p* < 0.001) [173]. In a study comprising 265 breast cancer patients receiving palliative chemotherapy, no significant difference was observed in the OS of diabetic versus non-diabetic subjects. However, OS was less in patients with hyperglycemia versus those not having a proper metabolic control (in both diabetic and non-diabetic groups). Moreover, a high mortality risk to cancer patients with glucose level of more than 130 mg/dL was observed [174]. A number of reports demonstrated that hyperglycemia enhances mortality of glioblastoma, non-small cell lung cancer, pancreatic cancer, breast cancer, hepatocellular carcinoma, gastric cancer, cervical cancer, esophageal cancer, endometrial, and colorectal cancer as shown in Supplementary Table S1.

Hyperglycemia associated risk factors affect the mortality and severity of the disease. A comprehensive literature survey investigating the effect of diabetes on any prognostic outcome in cancer patients compared with their nondiabetic counterparts was evaluated. Studies from MEDLINE, The Cochrane Library, CINAHL, and PsycINFO, which included patients with cancer and diabetes, were assessed. Cancer subjects with diabetes resulted in worse patient-reported outcomes (PRO) compared to having either one of the diseases [175]. 

## 6. Conclusions and Future Directions

Cancer is a disease of uncontrolled proliferation. The role of glucose, which functions as the primary source of energy, cannot be underestimated. The effect of variable extracellular levels of glucose on cancer cells is poorly understood. The recent resurgence in the diabetes–cancer link warrants further in-depth investigation. In diabetes, hyperglycemia, in particular, has been undervalued as a risk factor not only for cancer progression but also for disease development. Differential glucose uptake of cancer cells over normal cells is the primary mode of diagnosis through PET. Therefore, mechanisms by which cancer cells enhance glucose uptake, upregulate glycolysis and dysregulate the cell cycle can be strategically exploited for specifically targeting all types of cancers. The molecular events accompanying these are downstream effects of the former and play the secondary but essential role required to achieve important cancer characteristics such as immune evasion, apoptosis inhibition, etc. Since these effects vary according to the cancer type, the targets may be cell type specific. Hence, hyperglycemia-induced alterations serve as a model system for studying cancer metabolism and also for the discovery of a common therapeutic approach. This review presents a comprehensive study of hyperglycemia and its correlation with risk, progression, mortality, and outcome of various cancers to emphasize its role in all facets of cancer.

Hyperglycemia, irrespective of diabetes, obesity, or any other disorder, has pan effects on various organs throughout the body, causing global chaos via multiple metabolic and molecular mechanisms affecting DNA, RNA, and protein. DNA damage, DNA repair inhibition, mutation accumulation, activation of various pathways via lncRNA/miRNA, posttranslational modifications, rewiring energy metabolism, immune evasion, and chemoresistance are few of the effects resulting due to hyperglycemia on the biological system. Cellular alterations of hyperglycemia eventually lead towards adaptations, which if sustained, may cause worse consequences, resulting in conditions that favor or drive towards neoplastic transformation. 

In light of increasing lifestyle changes and growing pandemics of metabolic disorders, understanding how these alterations favor neoplastic transformation would not only help to reveal new druggable targets, but also to design a holistic approach towards prevention as well as treatment of this global epidemic. A coordinated effort of experts in metabolism, molecular biology, and pharmacology is needed for improving the understanding, prognosis, and better therapeutic outcomes in cancer patients.

## Figures and Tables

**Table 1:** Strategies to target cancer cells via glucose metabolism.

Target	Agent	Cancer	Phase	ReferenceNCT No./Pubmed ID
**GLUT1**	WZB117	NSCLC	H1299 and A549 (in vitro and in vivo)	22689530
		Breast	MCF-7 (in vitro and in vivo)	
		Breast	MCF-7/ADR resistant (in vitro)	28609310
		Breast	MDA-MB-231 and MCF-7	27011212
		Colon	5-FU-resistant human colon cancer cell line (in vitro)	25227787
		Neuroblastoma	SH-SY5Y (in vitro)	30553996
		Glioblastoma	Tumor-derived A172	29949049
	STF31	RCC	RCC4, Caki-1, SN12C (in vitro) 786-O and (in vitro and in vivo)	21813754
	Apigenin	Pancreatic cancer	CD18 and S2-013 pancreatic cancer cell lines	18953257
		Colon cancer	Phase 2 prevention of the recurrence of neoplasia	NCT00609310
	Genistein/Isoflavone G-2535	Hepatocellular carcinoma	HCC-LM3, SMMC7721, Hep3B, Bel-7402, and Huh-7	28926527
		Prostate	Phase 2	NCT00058266
		NSCLC	Phase 2	NCT01628471
		Colorectal	Phase 2	NCT01985763
		Prostate	Phase 3	NCT00584532
		Breast	Phase 2	NCT00290758
		Endometrial	Phase 1	NCT00099008
		Pancreatic	Phase 2	NCT00882765
		Bladder (I, II, III)	Phase 2	NCT00118040
		Kidney	Early Phase 1	NCT00276835
		Melanoma	Early Phase 1	NCT00276835
		Head and Neck	Phase 1	NCT02075112
		Leukemia	Phase 1	NCT00004858
		Lymphoma	Phase 1	NCT00004858
	Resveratrol/SRT501	Ovarian cancer	PA-1, OVCAR3, MDAH2774,SKOV-3	25307508
		Colon	Phase 1	NCT00256334
		Liver	Phase 2	NCT02261844
		Colon	Phase 1	NCT00433576
		Colorectal	Phase 1	NCT00920803
		Solid Tumor	Phase 1	NCT00098969
		Multiple Myeloma	Phase 2	NCT00920556
	Forskolin	Multiple Myeloma	H929 and OM-2	26306624
	Quercetin	Breast and ovarian cancer	MCF-7, MDA-MB-231, HBL100, BT549, OVCAR5, TOV112D, OVCAR3, CAOV3	26259240
**GLUT2**	Phloretin	Hepatocellular carcinoma	HepG2	19123483
**GLUT3**	Adriamycin and etoposide	Cervical and colon cancer	Hela and Caco-2 cell lines in vitro and in vivo	20870738
**GLUT4**	Ritonavir	Multiple myeloma	MM.1S and U266 cell lines	22452979
**GLUT5**	N-[4-(methylsulfonyl)-2-nitrophenyl]-1,3-benzodioxol-5-amine (MSNBA)	Breast cancer	MCF-7	27074918
**SLCA15**	GPNA, Benzylserine, γ-FBP, AOC, Chloroalanine		in silico	26444490, 29212300
**HK**	3-Bromopyruvate	Melanoma	in vivo	30206027
	2 Deoxy Glucose	Breast cancer	SKBR-3, MCF-7, MDA-MB-468, BT474	12232767
		Prostate cancer	Phase 2	NCT00633087
		Solid tumors	Phase 1	NCT00096707
	Lonidamine	Melanoma	DB-1 xenograft model	27497601
	3PO	Bladder cancer	in vitro	26504012
**PFK**	N4A	Lung cancer, colon cancer, pancreatic cancer	in vivo	23674815
		Breast Cancer	in vivo	18202014
		Breast Cancer, Cervical cancer	HeLa, T47D	21957443
**GAPDH**	Gossypol	Non-small Cell Lung Cancer	in vitro	30038571
		Non-small Cell Lung Cancer	in vitro	31055235
**PKM**	ML265	Lung cancer	in vitro	23905203
**LDH**	Oxamate	Renal cell carcinoma	in vivo	28983605
	FX11	Breast Cancer	in vivo	28243322
		Neuroblastoma	in vitro	27919448
		Prostate cancer	in vitro	25983002
	AT101 /Gossypol	Lymphoma	in vivo	20133848
		Prostate Cancer	Phase 2	NCT00666666
		Small Cell Lung Cancer	Phase 2	NCT00773955
	Galloflavin	Breast cancer	in vitro	22954722
		Endometrial cancer	in vitro	25631326
**PDH**	DCA	Oral squamous cell carcinoma	in vitro	25544754
		Head and Neck cancer	Phase 1	NCT01163487
	TT232	Melanoma	Phase 2	16393913
**MCT-4**	Phloretin	Breast cancer, prostate cancer, lymphoma	in vitro	27127175
**Complex 1**	Metformin	Colon cancer	in vitro and in vivo	24843020
	Rotenone	Leukemia	in vitro	12496265
	Piericidin	Breast cancer	in vitro	23690779
**Complex 2**	TTFA	Melanoma	in vitro	26521302
**Complex 3**	Stigmatellin	Lung cancer and Bone osteosarcoma	in vitro	17562787
	Myxothiazol	Colon cancer	in vitro	24772329

**Table 2:** Anti-hyperglycemic/anti-diabetic drugs alone or in combination for cancer therapy.

Cancer	Combination	Phase	NCT No:
Metastatic colorectal cancer	Metformin, 5-Fluorouracil	Phase 2	NCT01941953
Her2 positive breast cancer	Liposomal Doxorubicin, Docetaxel, Trastuzumab, Metformin	Phase 2	NCT02488564
Breast cancer	Metformin, Doxorubicin	Phase 2	NCT02472353
Human epidermal growth factor 2 negative carcinoma of breast	Metformin, Myocet, Cyclophosphamide	Phase 2	NCT01885013
	Myocet + Cyclophosphamide		
Diffuse large B-cell lymphoma	Metformin, Rituximab, Cyclophosphamide, Doxorubicin, Vincristine, Prednisone, Pegfilgrastine	Phase 2	NCT02531308
Lung cancer, Breast cancer, Pancreatic cancer, Head and neck cancer, Gastric cancer	2-Deoxyglucose, Docetaxel	Phase 1	NCT00096707
Pancreatic cancer	Capecitabine, Cisplatin, Epirubicin, Gemcitabine, Metformin	Phase 2	NCT01167738
Prostate cancer	Rosiglitazone and Placebo	Phase 3	NCT00182052
Pancreatic cancer	Pioglitazone	Phase 2	NCT01838317

## Supplementary Materials

The following are available online at https://www.mdpi.com/2072-6694/11/9/1402/s1, Figure S1: Metabolic disorders and cytokine signaling in cancer, Figure S2: Combinatorial effect of hyperglycemia associated alterations in cancer, Table S1: Increased mortality of different cancers under hyperglycemic/diabetic conditions.
