# Supplementary material for: Hyperglycemia Associated Metabolic and Molecular Alterations in Cancer Risk, Progression, Treatment, and Mortality
Source: Cancers (Basel). 2019 Sep 19;11(9):1402. doi: 10.3390/cancers11091402 (PMC6770430; doi:10.3390/cancers11091402)
Supplement: Supplementary file 1 [file cancers-11-01402-s001.pdf]

cancers

MDPI

Supplementary Material

Hyperglycemia Associated Metabolic and Molecular Alterations in Cancer Risk, Progression, Treatment, and Mortality

Pranay Ramteke, Ankita Deb, Varsha Shepal and Manoj Kumar Bhat

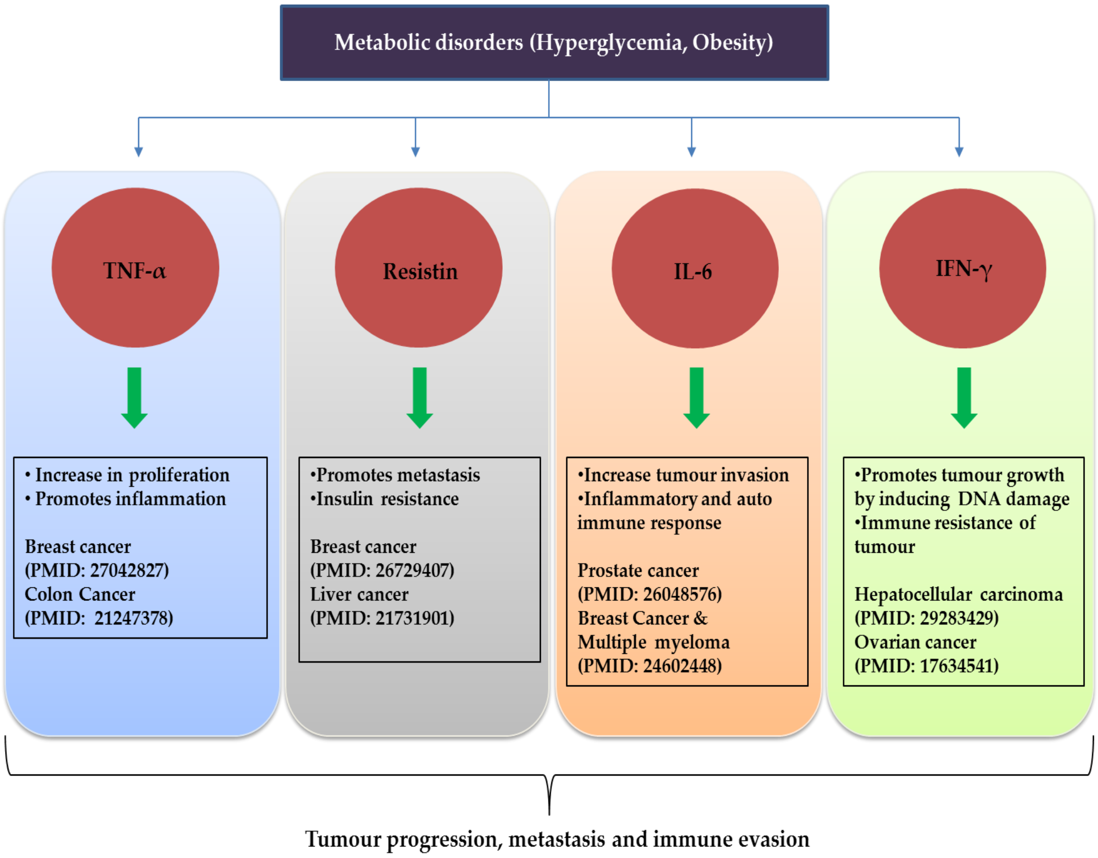

Figure S1. Metabolic disorders and cytokine signaling in cancer.

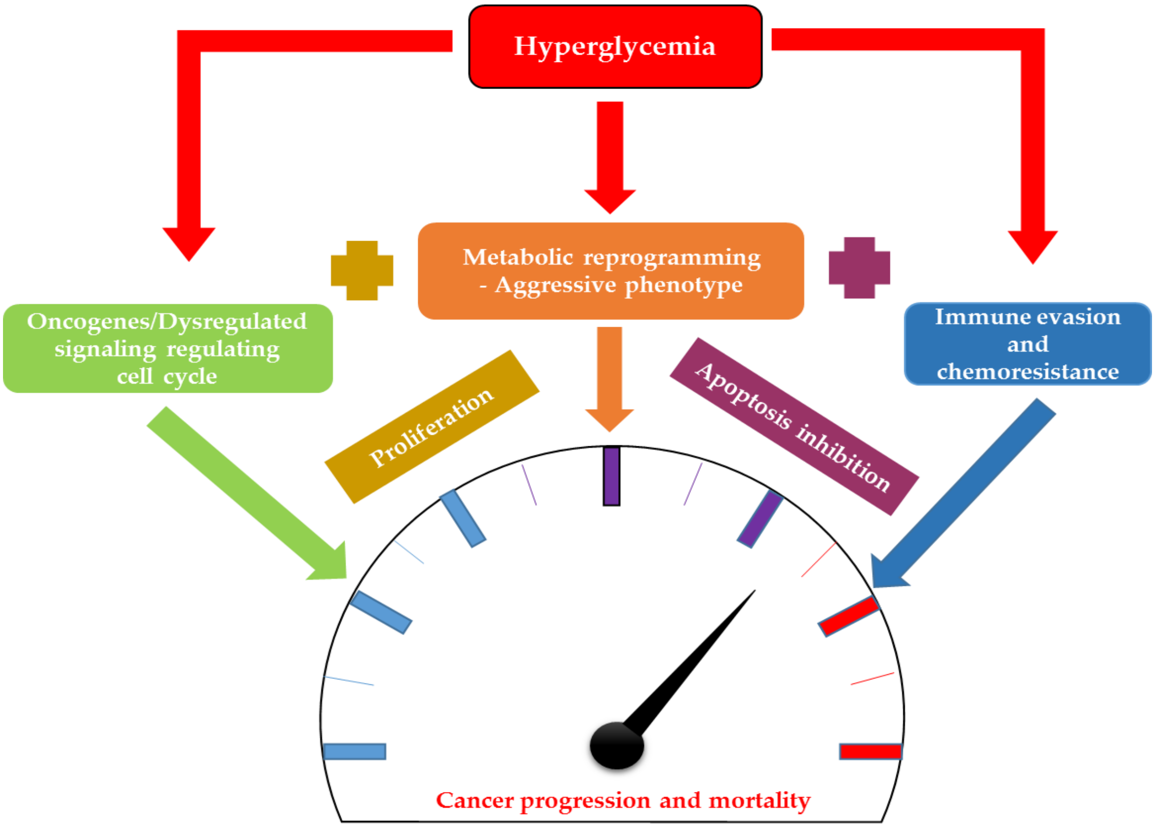

Figure S2. Combinatorial effect of hyperglycemia associated alterations in cancer.

Table S1. Increased mortality of different cancers under hyperglycemic/diabetic conditions.

Reference	Cancer	Hazard ratio/Mortality rate (%)
Adeberg et.al., 2016 [1]	Glioblastoma	2.44
Arrieta et.al., 2016 [2]	Non-small cell lung cancer	2.04
Cheon et.al., 2014 [3]	Pancreatic cancer	2.55
Erickson et. al., 2011 [4]	Early stage breast cancer	2.35
Hosokawa et.al., 2013 [5]	Hepatocellular carcinoma	2.77
Hu et.al., 2017 [6]	Gastric cancer	1.53
Li et.al., 2017 [7]	Cervical cancer	4.35
Okamura et.al., 2017 [8]	Esophageal cancer	1.72
Duan et.al., 2014 [9]	Endometrial, breast and colorectal cancer	41%

References

1. Adeberg, S.; Bernhardt, D.; Foerster, R.; Bostel, T.; Koerber, SA.; Mohr, A.; Koelsche, C.; Rieken, S.; Debus J. The influence of hyperglycemia during radiotherapy on survival in patients with primary glioblastoma. *Acta Oncol*. **2016**, *55*, 2017.
2. Arrieta, O.; Varela-Santoyo, E.; Soto-Perez-de-Celis, E.; Sánchez-Reyes, R.; De la Torre-Vallejo, M.; Muñiz-Hernández, S.; Cardona, A.F. Metformin use and its effect on survival in diabetic patients with advanced non-small cell lung cancer. *BMC Cancer* **2016**, *16*, 633. doi: 10.1186/s12885-016-2658-6.
3. Cheon, Y.K.; Koo, J.K.; Lee, Y.S.; Lee, T.Y.; Shim, C.S. Elevated hemoglobin A1c levels are associated with worse survival in advanced pancreatic cancer patients with diabetes. *Gut Liver* **2014**, *2*, 205–214.
4. Erickson, K.; Patterson, R.E.; Flatt, S.W.; Natarajan, L.; Parker, B.A.; Heath, D.D.; Laughlin, G.A.; Saquib, N.; Rock, C.L.; Pierce, JP. Clinically defined type 2 diabetes mellitus and prognosis in early-stage breast cancer. *J. Clin. Oncol*. **2011**, *29*, 54–60.
5. Hosokawa, T.; Kurosaki, M.; Tsuchiya, K.; Matsuda, S.; Muraoka, M.; Suzuki, Y.; Tamaki, N.; Yasui, Y, Nakata, T.; Nishimura, T.; et. al. Hyperglycemia is a significant prognostic factor of hepatocellular carcinoma after curative therapy. *World J. Gastroenterol*. **2013**, *19*, 249–257. doi: 10.3748/wjg.v19.i2.249.

6. Hu, D.; Peng, F.; Lin, X.; Chen, G.; Zhang, H.; Liang, B.; Ji, K.; Lin, J.; Chen, LF.; Zheng, X.; Niu, W. Preoperative Metabolic Syndrome Is Predictive of Significant Gastric Cancer Mortality after Gastrectomy: The Fujian Prospective Investigation of Cancer (FIESTA) Study. *EBioMedicine* **2017**, *15*, 73–80.
7. Li, J.; Nin,g N.Y.; Rao, Q.X.; Chen, R.; Wang, L.J.; Lin, Z.Q. Pretreatment glycemic control status is an independent prognostic factor for cervical cancer patients receiving neoadjuvant chemotherapy for locally advanced disease. *BMC Cancer* **2017**, *17*, 517. doi: 10.1186/s12885-017-3510-3.
8. Okamura, A.; Watanabe, M.; Imamura, Y.; Hayami, M.; Yamashita, K.; Kurogochi, T.; Mine, S. Glycemic Status and Prognosis of Patients with Squamous Cell Carcinoma of the Esophagus. *World J. Surg.* **2017**, *41*, 2591–2597. doi:10.1007/s00268-017-4036-1.
9. Duan, W.; Shen, X.; Lei, J.; Xu, Q.; Yu, Y.; Li, R.; Ma, Q. Hyperglycemia, a neglected factor during cancer progression. *BioMed. Res. Int.* **2014**, *2014*, 461917. doi:10.1155/2014/461917.
